# Lipidated DAPEG Polymers as a Non-Toxic Transfection Agent—Influence of Fatty Acid Side Chain on Transfection Efficacy

**DOI:** 10.3390/molecules30071644

**Published:** 2025-04-07

**Authors:** Wiktoria Mallek, Anita Romanowska, Wiktoria Machowicz, Agnieszka Piwkowska, Adam Lesner, Magdalena Wysocka

**Affiliations:** 1 Faculty of Chemistry, University of Gdansk, Wita Stwosza 63, 80-308 Gdansk, Poland; wiktoria.mallek@phdstud.ug.edu.pl (W.M.); anita.romanowska@ug.edu.pl (A.R.); w.machowicz.912@studms.ug.edu.pl (W.M.); 2 Mossakowski Medical Research Institute Polish Academy of Sciences, Wita Stwosza 63, 80-308 Gdansk, Poland; apiwkowska@imdik.pan.pl

**Keywords:** peptidomimetics, transfection, lipidated polymers

## Abstract

This study describes the synthesis, interaction with DNA, and transfection efficacy of eight lipidated compounds based on a recently published non-lipidated parent molecule, an octamer of 2,3-l-Dap, carrying the guanidine group on its side chain. The compounds obtained were found to be non-toxic up to 5 µM and efficient DNA binders and showed greater transfection efficiency than the parent compound, with two leading molecules containing acetic and decanoic moieties. DLS experiments indicated two groups of interaction with DNA. One group modified by short-chain lipids (up to eight carbon atoms in the main chain) forms large structures due to the aggregation of multiple nucleic acids. The second group (from twelve to sixteen carbon atoms) with dominant condensation creates smaller forms and is less effective in transporting DNA into the cells.

## 1. Introduction

### Transfection

Introducing nucleic acid into cells is a key therapeutic approach in modern medicine. This process is crucial to proteins, vaccines (such as the COVID-19 vaccine), and drug production, as well as in medical diagnostics [1,2]. There are several examples of proteins produced in HEK293 cell line approved by FDA like NUWIQ (recombinant anti-hemophilic factor) or multiple antibody entities [3].

There are several methods of nucleic acid delivery, categorized into viral and non-viral transfections. Among the non-viral transfection methods, chemical reagents like polybasic compounds or peptides, as well as hydrophobic or amphipathic polymers are widely used [4,5]. The most frequently used group of transfection reagents are lipidated charged molecules consisting of a cationic residue, typically an amine or amino acid (Arg, Lys) and at least one carboxylic acid with a relatively long carbon chain attached to its functional group [6]. These compounds display amphiphilic properties with a positively charged hydrophilic area that interacts with the phosphonic groups of nucleic acids and a hydrophobic side chain that interacts with the non-polar region of cell membranes. The hydrophobic portion of the molecule plays a crucial role in determining the structure of the complex formed and the transfection efficacy [7]. These compounds have been reported in several studies to exhibit low cytotoxicity and are efficient transfection reagents that transport different forms of nucleic acid (plasmids, siRNA, or others). Cullis and Felgner provide an excellent review of this group of compounds [8].

A few years ago, our research group introduced some novel building blocks that mimic amino acid residues. These building blocks consist of L-2,3-diaminopropionic acid substituted on its side chain by different functionalized oxa acids (short ethylene glycol monomers) [9]. Due to the variety of functional side chain groups, these molecules could serve as an alternative to amino acid residues with easily modifiable side chain lengths. Using the described building blocks, further referred to as DAPEG, we were able to construct libraries of labeled peptidomimetics. Deconvolution of these libraries yielded potent substrates of physiologically significant proteases like NSP4 or the trypsin subunit of the 20 S proteasome [9,10].

**Figure 1:**
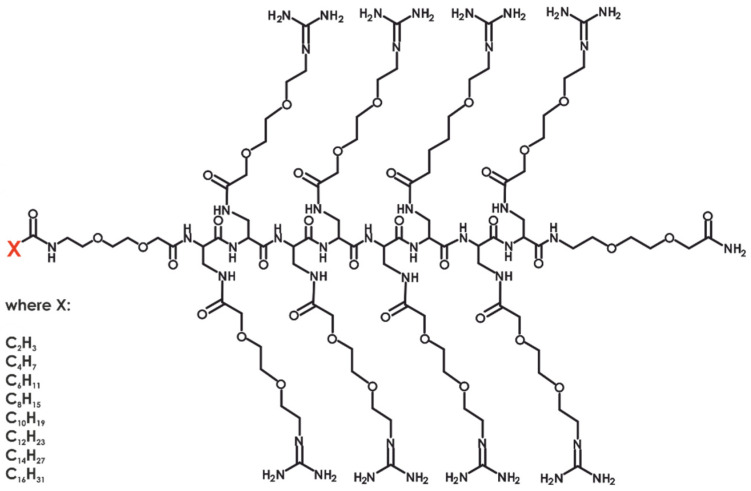
General formula of N-terminal lipidated polymers.

Recently, we synthesized a series of compounds using the general formula shown in Figure 1 and demonstrated that some of them are effective mediators of transfection of a GFP-coding plasmid into both cancerous and healthy cell lines [11]. These synthesized compounds contain eight DAPEG residues that mimic an Arg residue, each with different side chain lengths and hydrophobicity due to the presence of oxygen atoms within them.

Additionally, we have shown that when these compounds are biotinylated, they can form a complex with Strp-β-gal conjugate and act as universal carriers for delivering active forms of the enzymes [12].

In this work, we developed a series of lipid N-modified analogs of the best compounds identified in our previous study as potential transfection reagents. We employed eight different carboxylic acids (acetic, butanoic, hexanoic, octanoic, decanoic, dodecanoic, tetradecanoic, and hexadecenoic) to synthesize eight compounds. The physicochemical properties of these compounds are outlined in Table 1.

To evaluate transfection efficiency, we used the spheroid HEK-293T cell line. This cell line was frequently employed in various expression systems and has been extensively discussed in paper [13].

The DNA plasmid encoding the fluorescent protein (GFP), p_max_GFP, was used as a model for gene transfer and expression.

## 2. Results and Discussion

Following the previously described synthetic step, we obtained eight *N*-modified analogs of the parent compound. All compounds were characterized using physicochemical methods, including UPLC and HR-MS (see Table 1) and desalted.

**Figure 2:**
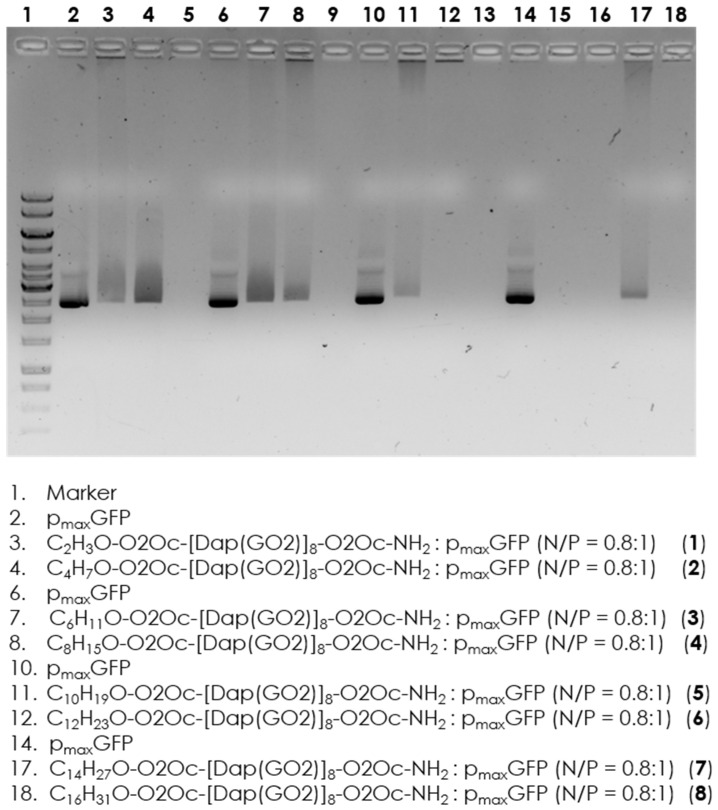
Electrophoretic separation of p_max_GFP mixed with a variety of lipidated compounds. Line **1** contains the loaded marker (GeneRuler 1 kb DNA ladder). Lines 5, 9, 13, 15, and 16 are intentionally left empty. Lines 2, 6, 10, and 14 contain only the plasmid. Lines 3, 4, 7, 8, 11, 12, 17, and 18 show a mixture of p_max_GFP with the appropriate compound (**1**,**2**,**3**,**4**,**5**,**6**,**7**,**8**), respectively.

Since we have data on the efficient DNA binding to the parent compound (**1a**) [11], we determined to verify whether the attachment of fatty acids would disrupt these interactions. Electrophoretic separation of trhe formed complexes with implementation of the appropriate controls was performed. The results are shown in Figure 2. Evidence shows that all compounds can interact with DNA at a concentration of 2 µM. Therefore, we can proceed with the next step of selecting the transfection reagent, which involves separately incubating the GFP coding plasmid with the obtained compounds.

As shown in Figure 2, a shift in migration between the plasmid marker and the complex is observed in all wells, indicating effective interaction of all compounds with the model DNA. There is no significant difference between the individual systems where the tested compounds are present.

**Figure 3:**
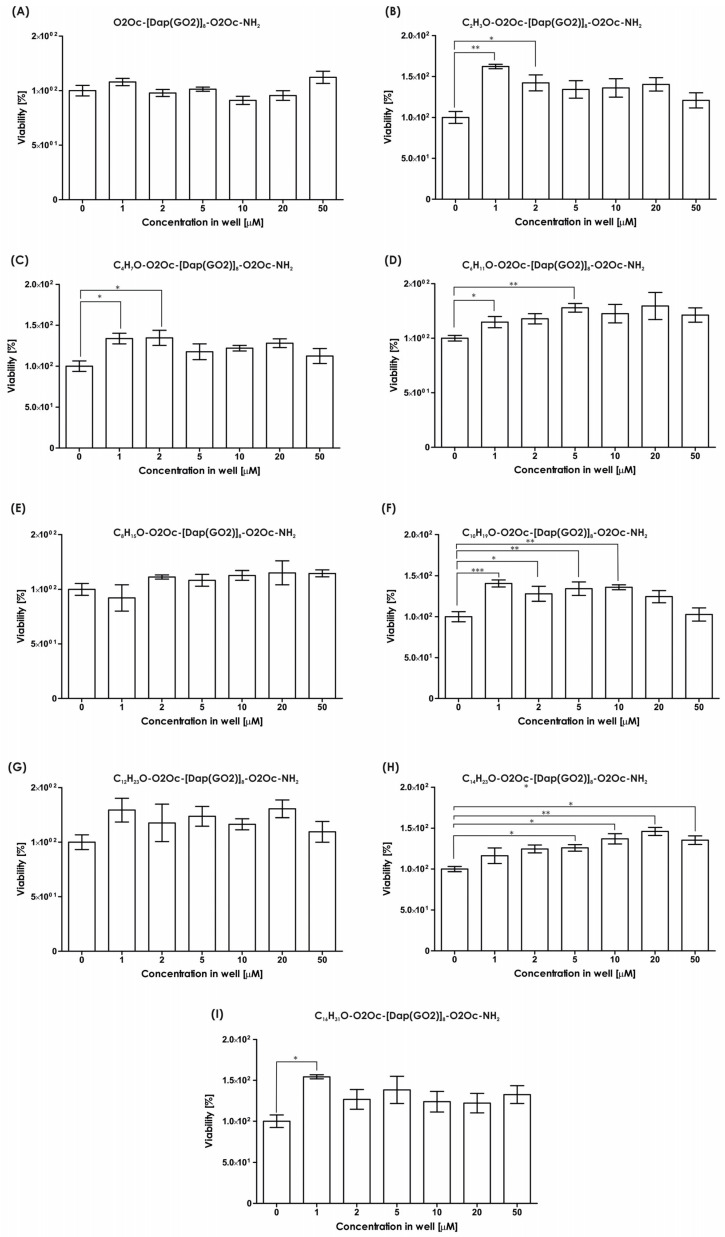
The cytotoxicity assay results of the synthesized compounds (mean ± SD; n = 3). Statistical calculations were performed using one-way ANOVA: * *p* < 0.05; ** *p* < 0.01; *** *p* < 0.001 (number of samples in one system n = 3). (**A**) Cytotoxicity assay of H-O2Oc-[Dap(GO2)]_8_-O2Oc-NH_2_; (**B**) cytotoxicity assay of C_2_H_3_O-O2Oc-[Dap(GO2)]_8_-O2Oc-NH_2_; (**C**) cytotoxicity assay of C_4_H_7_O-O2Oc-[Dap(GO2)]_8_-O2Oc-NH_2_; (**D**) cytotoxicity assay of C_6_H_11_O-O2Oc-[Dap(GO2)]_8_-O2Oc-NH_2_; (**E**) cytotoxicity assay of C_8_H_15_O-O2Oc-[Dap(GO2)]_8_-O2Oc-NH_2_; (**F**) cytotoxicity assay of C_10_H_19_O-O2Oc-[Dap(GO2)]_8_-O2Oc-NH_2_; (**G**) cytotoxicity assay of C_12_H_23_O-O2Oc-[Dap(GO2)]_8_-O2Oc-NH_2_; (**H**) cytotoxicity assay of C_14_H_27_O-O2Oc-[Dap(GO2)]_8_-O2Oc-NH_2_; (**I**) cytotoxicity assay of C_14_H_31_O-O2Oc-[Dap(GO2)]_8_-O2Oc-NH_2_.

The impact of the resulting compounds on cells was evaluated using the MTT assay, and the results are illustrated in Figure 3. The MTT test measures the activity of mitochondria to determine the number of living cells. As indicated in Figure 3, none of the tested compounds exhibited any toxic effects in the tested concentration range up to 50 µM. Conversely, in all systems, the presence of polymers stimulates cell growth, with a statistically significant impact observed (see Figure 3).

**Figure 4:**
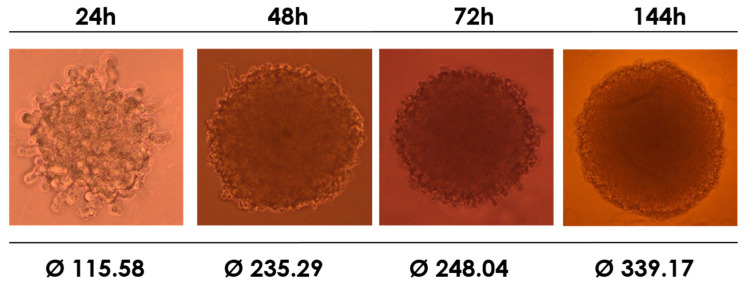
Visible pictures of spheroid formation of the HEK-293T cell line at four different periods. The diameter values are expressed in µm.

Next, we investigated the viability of the synthesized compounds against the HEK-293T cell line using cell culture in a 3D model. The cells were able to form the expected structures in a time-dependent manner. As seen in Figure 4, the spheroids were fully formed within 72 h. It is widely acknowledged that a system that forms spheroid cells better reflects the natural environment and condition of cell growth than single-layer culturing.

**Figure 5:**
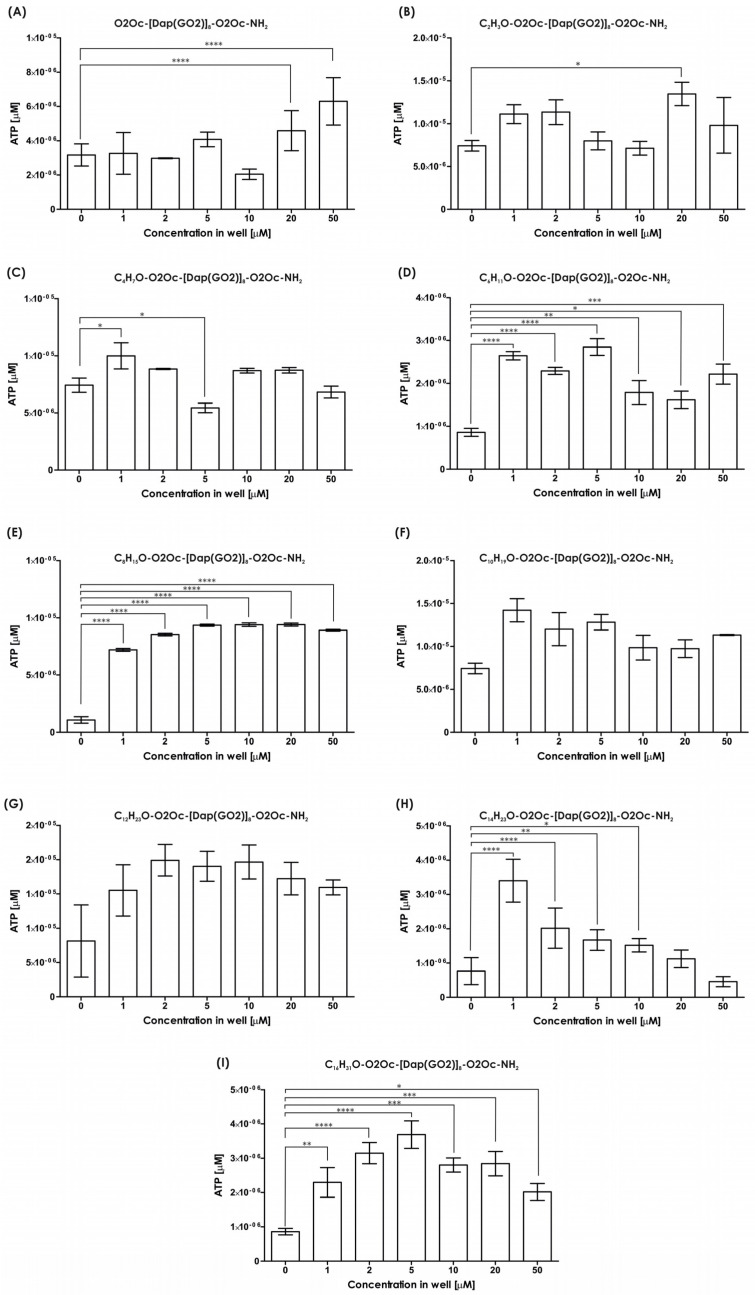
Viability of lipidated compounds against the HEK-293T cell line (mean ± SD; n = 3). Statistical calculations were performed using one-way ANOVA: * *p* < 0.05; ** *p* < 0.01; *** *p* < 0.001; **** *p* < 0.0001 (number of samples in one system n = 3). (**A**) Viability of H-O2Oc-[Dap(GO2)]_8_-O2Oc-NH_2_ against the HEK-293T; (**B**) viability of C_2_H_3_O-O2Oc-[Dap(GO2)]_8_-O2Oc-NH_2_ against the HEK-293T; (**C**) viability of C_4_H_7_O-O2Oc-[Dap(GO2)]_8_-O2Oc-NH_2_ against the HEK-293T; (**D**) viability of C_6_H_11_O-O2Oc-[Dap(GO2)]_8_-O2Oc-NH_2_ against the HEK-293T; (**E**) viability of C_8_H_15_O-O2Oc-[Dap(GO2)]_8_-O2Oc-NH_2_ against the HEK-293T; (**F**) viability of C_10_H_19_O-O2Oc-[Dap(GO2)]_8_-O2Oc-NH_2_ against the HEK-293T; (**G**) viability of C_12_H_23_O-O2Oc-[Dap(GO2)]_8_-O2Oc-NH_2_ against the HEK-293T; (**H**) viability of C_14_H_27_O-O2Oc-[Dap(GO2)]_8_-O2Oc-NH_2_ against the HEK-293T; (**I**) viability of C_14_H_31_O-O2Oc-[Dap(GO2)]_8_-O2Oc-NH_2_ against the HEK-293T.

All compounds underwent viability assays using 3D models. The amount of ATP was measured as a marker of living cells. We tested all compounds to determine the effect of lipidation. Several reports strongly suggest that lipidation of peptides generates more toxic molecules compared to non-modified compounds. The spheroids of the HEK-293T cells were incubated with the compounds mentioned above and cultured for 24 h. The results of the viability assay are provided in Figure 5. The viability of the tested cells exposed to each compound (Figure 5A–I) are not suppressed compared to the non-treated cells up to 10 µM, except for compound **1,** with an acetyl moiety, which is not toxic up to 5 µM. No typical dose–response effect is observed except in Figure 5H. The introduction of short-chain fatty acids (up to system D, which corresponds to hexanoic acid) results in a wave-like shape of the live cell number with a minimum dose of around 5 or 10 µM and two maxima at 1–2 µM and 20–50 µM, where stimulation of cell growth is observed. The presence of longer-chain fatty acids (Figure 5E–I, except for 5H as mentioned above) strongly induces spheroid growth, with the number of cells exceeding the non-treated control several times. In conclusion, all the compounds did not suppress cell growth up to 5 µM. The effect of increased cell proliferation is not anticipated and must be considered when utilizing such compounds in living organisms. Uncontrolled stimulation of cell growth is a risk factor for cancer. However, in cell culture, increased growth of transfection-resistant cells appears to be advantageous, as it increases the yield of the final transfection product, i.e., recombinant proteins.

**Figure 6:**
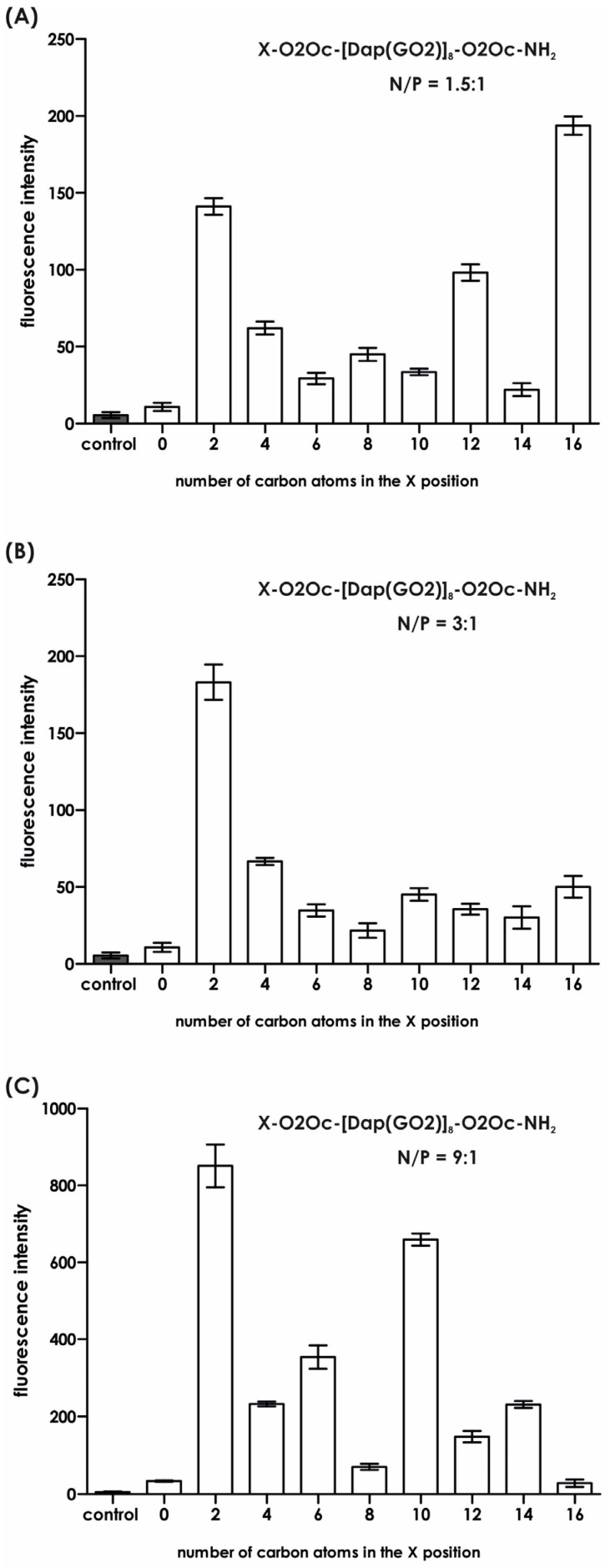
The impact of fatty acid length attached to the parent compound on the transfection efficacy of GFP plasmid into the HEK-293T cell line (mean ± SD; n = 3). (**A**) Transfection efficiency at a N/P ratio of 1.5:1; (**B**) transfection efficiency at a N/P ratio of 3:1; (**C**) transfection efficiency at a N/P ratio of 9:1.

**Figure 7:**
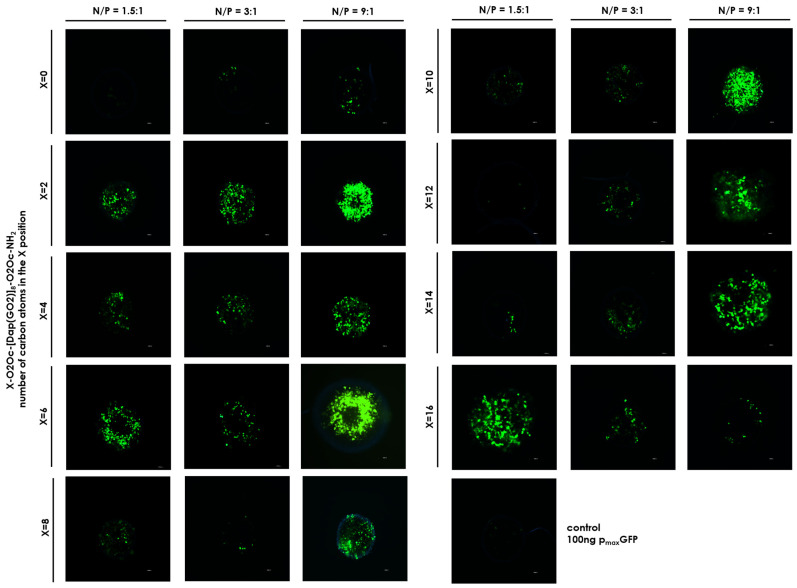
Confocal fluorescence microscopy images of the HEK-293T cells incubated with complexes. Magnification 20× scale bar 10 µm.

Since we found no toxic effects from the obtained compounds, we proceeded to the next stage of our research and performed the key experiment, which involved transfecting a model plasmid into the HEK-293T cell line. It is important to note that the maximum concentration of the compounds used never exceeded 5 µM, a concentration deemed safe for the cells used. The results of the GFP production efficacy are shown in Figure 6 and Figure 7. Figure 6 was generated based on the mean fluorescence of the spheroids visible in Figure 7. Significant differences were observed for all compounds in each system depending on the N/P ratio used in the experiment. For the system with the lowest N/P ratio of 1.5:1, the greatest fluorescence (due to GFP production) was observed for compound **8,** followed by compound **1**, with an average value in the range of 150 arbitrary units (Figure 5A). When we doubled the N/P ratio (Figure 6B), compound **1** became the highest with an average fluorescence of around 200. None of the other compound complexes with the plasmid exhibited at least half of the fluorescence observed in compound **1**. For N/P = 9:1, which is six times the value of N/P = 1.5:1, superior fluorescence was observed for compound **1**, followed by compounds **3** and **5**. The other compounds did not induce significant amounts of GFP (Figure 6 and Figure 7). It is noteworthy that the transfection efficacy of compound **1a,** the parent compound with three N-terminal amino groups, is the lowest in each series. This underscores the importance of masking a positive charge in this part of the molecule. We do not have a reasonable explanation for why a particular substituent strongly affects the transfection efficacy. In order to further illuminate this issue, an investigation was conducted into the stability of the formed complex under conditions that were identical to those used for the transfection, including the reagent buffer composition and the transfection period. Subsequently, at predetermined time points, UV spectra were recorded for each system, in addition to the controls, and are presented in Figure S2. An analysis of the obtained results indicates that the spectra properties of the lipidated compound alone and in a complex with DNA are quite similar to each other, except for compounds **4** and **1**, which do not show significant changes over time. For all other compounds, we observed a decrease in intensity over time.

**Figure 8:**
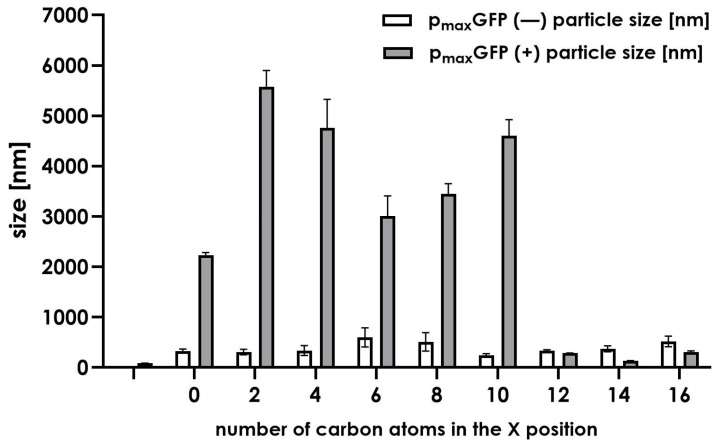
DLS results of series of lipidated compounds—DNA plasmid complex (mean  ±  SD; n  =  3).

**Figure 9:**
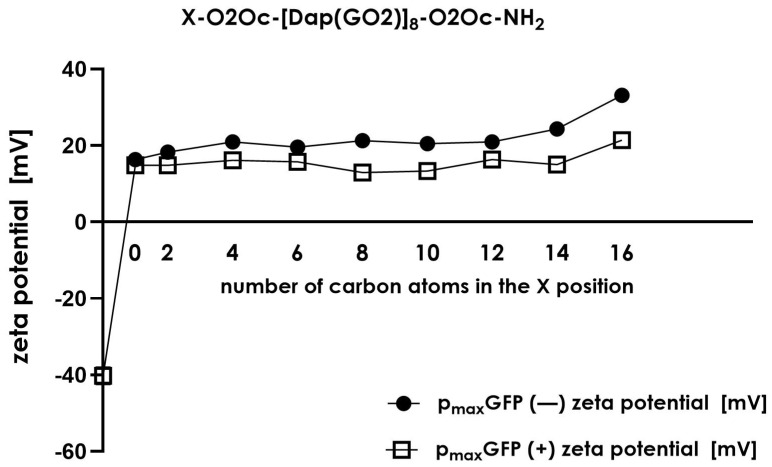
The zeta potential of lipidated compounds (black dot) and their complexes with p_max_GFP (empty square) (mean  ±  SD; n  =  3).

To thoroughly investigate the nature of such discrepancies, we conducted additional experiments to understand the formation of complexes between DNA and the tested compounds. We used dynamic light scattering (DLS) to determine whether the formed complexes are uniformly organized in terms of their size (Figure 8). Additionally, we measured the zeta size of each complex to confirm the electrostatic nature of the compound–plasmid interactions (Figure 9).

Initially, we conducted several experiments using DLS to provide an overview of the average particle size of the compounds with and without model DNA plasmid. As shown in Figure 8, the sizes of the particles formed by the tested compounds mostly range between 140 and 600 nm, indicating the presence of liposomes formed by synthesized molecules. Upon the addition of plasmid DNA (p_max_GFP), larger structures were observed. In systems **1a** to **6** (corresponding to compounds **1**–**5** along with a control lacking N-terminal modification), large diameter particles ranging from 2000 to almost 6000 nm were recorded. We believe this is an aggregation effect resulting from the interaction between multiple plasmids and compound instances. No large structures were observed in compounds **6**–**8**. The compounds alone displayed average particle sizes ranging between 300 and 600 nm. The addition of plasmid p_max_GFP caused the particle sizes to be reduced to the range of 200–400 nm. This observation suggests the condensation of DNA or DNA packing into a liposome-like structure.

Zeta size measurements provide information about the overall charge carried by the formed particles. As shown in Figure 9, we observed a change in charge when the plasmid was incubated with the selected compound. In each case, the complex formed displayed a positive charge in contrast to the negative charge of the plasmid. The zeta size of particles in systems without DNA in the mixture displayed the greatest values (18–35 mV) compared to the charge of complexes formed in the presence of plasmid DNA (15–20 mV). This indicates that in the presence of plasmid DNA, the zeta size of all structures present in the solution reduces their charges by up to 20–30%, showing condensation or stabilization of formed particles compared to systems lacking DNA. However, the overall charge in all conditions was positive except for plasmid DNA, which was expected to be negative.

Our results are consistent with several reports indicating that introducing lipids into a polyArg sequence (preferably octamer) is an effective method for delivering various types of nucleic acids into the desired cells [14,15,16]. To the best of our knowledge, there has been no comprehensive study on the effect of fatty acid chain length on the transfection efficacy of cationic lipids containing DAPEG residues. Similar research has been conducted on Arg-rich peptides [17] or other cell-penetrating peptides (CPPs) like PeptFECT14 (an analog of transportan [18]). Previous studies on a series of fatty acid-modified peptides have shown moderate to severe toxicity, reaching 50% at a concentration of 10 µM for fatty acids with carbon chains longer than 12 [19]. This toxic effect is associated with increased transfection efficiency, probably due to the partial damage of the cell membrane. However, we did not observe such drastic changes since the tested compounds were non-toxic up to a concentration of 20 µM while still maintaining effective transfection capabilities.

## 3. Materials and Methods

### 3.1. Synthesis

All compounds were synthesized according to the previously described procedure [11]. Following the final Fmoc-removal, the N-terminal lipid attachment was performed using a two-fold excess of the corresponding carboxylic acid. The compounds obtained were then detached from the resin using a mixture of TFA, phenol, water, thioanisole, and EDT (82.5:5:5:5:2.5 *v*/*v*), lyophilized, and analyzed using RP UPLC (Shimadzu, Kyoto, Japan, software: LabSolutions LC/GC 5.54 SP2) and HR MALDI MS (Bruker Daltonics GmbH & Co. KG, Bremen, Germany, software: Compass for flexSeries 1.4).

### 3.2. Purification

All compounds were desalted using Speedisk^®^ C8SPE column (BAKERBOND, Chihuahua, TX, USA). The 10 mg/mL solution was applied on the column wash by water: methanol (95:5, *v*/*v*) and eluted by water: methanol 85:15 (*v*/*v*) mixture. The obtained solution was evaporated dissolved in water and lyophilized.

### 3.3. DNA Interactions

The individual compounds **2**–**9** were mixed at a concentration of 5 × 10^−5^ M with 100 ng of plasmid DNA and then incubated at 24 °C. Four microliters of the GeneRuler 1 kb DNA ladder (Thermo Scientific, Waltham, MA, USA) were used. A 0.7% agarose gel was prepared (UltraPure Agarose Thermo Fisher, Osterode Am Harz, Germany) and run at 150 V for 55 min in a TAE buffer. The MidoriGreen (advanced DNA stain, Nippon Genetics Europe GmbH, Düren, Germany) was applied to visualize the DNA.

### 3.4. Cell Culture

The HEK-293T cell line was cultured using both 2D and 3D techniques. The non-adherent method of forming spheroids was used. Cells (750 cells per well) were seeded in a 96-well U-shaped plate BIOFLOAT (Sarstedt, Nümbrecht, Germany). The HEK-293T cells were cultured at 37 °C in 5% CO_2_ in Dulbecco’s modified Eagle medium (DMEM; high glucose) supplemented with 10% fetal bovine serum (FBS) and 1% penicillin–streptomycin solution containing 100 units of penicillin and 100 µg/mL of streptomycin. After three days of culturing, the spheroids were formed and ready for further experiments.

### 3.5. Cytotoxicity

The classical MTT test was used. After 72 h of culturing, the HEK-293T cells (8000 cells per well of a 96-well plate) were incubated with increasing amounts of the appropriate compounds (0, 1, 2, 5, 10, 20, and 50 µM) in the medium described above at 37 °C with 5% CO_2_. All procedures were performed as outlined in [11].

### 3.6. Viability

After culturing for 72 h, spheroids of HEK-293T cells were incubated with increasing amounts of the appropriate compounds (0, 1, 2, 5, 10, 20, and 50 µM) in the medium described above at 37 °C with 5% CO_2_. After 24 h, the plate was incubated at room temperature (RT) for 30 min, followed by the addition of 100 μL of CellTiter Glo 3D Cell Viability Assay reagent (Promega, Fitchburg, WI, USA). The plate was then covered from light and shaken for 30 min. Next, the contents of each well were transferred to a white flat bottom plate (Nunc A/S, Thermo Fisher, Odense, Denmark) and luminescence was read at 560 nm using the CLARIOstar (BMG Labtech, Ortenberg, Germany).

### 3.7. Transfection Efficacy in 3D Model

Cells were cultured for 72 h under the conditions described above. Mixtures of the selected compounds and p_max_GFP plasmid in N/P ratios (charge peptidomimetic/charge p_max_GFP) of 1.5, 3:1, and 9:1 were incubated for 30 min and used immediately. An appropriate control with plasmid was employed. To each well with spheroids, 10 µL of the mixture described above was added in triplicate up to 90 µL of the full medium. The plate was then incubated at 37 °C with 5% CO_2_ for 5 h, washed (3 × medium), and cultured for another 72 h. After this process, the cells were inspected under a fluorescent microscope (Olympus IX51 fluorescence microscope (Olympus, Tokyo, Japan)). The molar concentration of particular components was equal to, for system 1:1.5, the concentration of each compound at 0.61 µM; for 3:1–1.21 µM; for 9:1–3.64 µM. The DNA plasmid concentration remains constant and equal at 0.46 nM.

The time-dependent transfection efficiency was tested for the selected compounds at 1.5, 3, and 5 h time points. The procedure was the same as described above.

### 3.8. DLS Zeta Size Measurements

Dynamic light scattering (DLS) measurements were performed using the Litesizer DIA 500 (Anton Par, Ostfildern-Scharnhausen, Germany). The concentration of the compounds in water was 2.5 mg/mL, with the inclusion of a DNA plasmid to maintain a charge-to-ratio of 1.5/1 N/P. For system 1:1.5, the concentration of each compound was 0.61 µM; for 3:1–1.21 µM; for 9:1–3.64 µM. The DNA plasmid concentration remains constant and equal at 0.46 nM. The particle size series mode was used in a cuvette with a volume of one quart. Each run was recorded at 25 °C for 10 s.

### 3.9. Zeta Size Measurement

Zeta sizing was performed using the same apparatus mentioned above. The concentration of each compound in water with DNA complexes was consistent with the DLS measurements (Anton Paar GmbH, Graz, Austria). The Univette low-volume cuvette was used. Voltage was adjusted automatically.

### 3.10. Complex Stability Assessment

The analysis of all obtained complexes was conducted using a Nanodrop 2000c UV-Vis spectrophotometer (Thermo Scientific, Waltham, MA, USA) at designated time points from 0 to 5 h (0, 0.5, 1, 2.5, and 5). The concentration of the individual components (DNA and the compound) was used as a reference. The concentration of the complexes remained constant in relation to the transfection experiments.

## 4. Conclusions

In this work, we have confirmed that lipidation of positively charged molecules increases transfection efficacy in the majority of systems compared to non-modified parent compounds. Surprisingly, no toxic effects resulted from the introduction of lipids. DLS experiments indicate two groups of interactions with DNA: One group modified by short-chain lipids (up to ten carbon atoms in the main chain) forms large structures due to the aggregation of multiple nucleic acids. The second group (from twelve to sixteen carbon atoms) with dominant condensation creates smaller forms, resulting in less effective DNA transport into the cells. We believe that such molecules, considering their low cytotoxicity and efficient transfection efficacy, could be an attractive solution for introducing nucleic acids into cells.

## Figures and Tables

**Table 1:** Physicochemical characteristics of synthesized compounds.

No	Sequence	Retention Time * [min]	Molecular Weight Calculated/ Determined **	Yield *** %
**1a.**	H-O2Oc-[Dap(GO2)]_8_-O2Oc-NH_2_ [10]	-	-	
**1**	C_2_H_3_O-O2Oc-[Dap(GO2)]_8_-O2Oc-NH_2_	4.87	2533.81/2535.22	64
**2**	C_4_H_7_O-O2Oc-[Dap(GO2)]_8_-O2Oc-NH_2_	5.06	2561.81/2563.30	53
**3**	C_6_H_11_O-O2Oc-[Dap(GO2)]_8_-O2Oc-NH_2_	5.45	2589.86/2591.36	68
**4**	C_8_H_15_O-O2Oc-[Dap(GO2)]_8_-O2Oc-NH_2_	5.78	2617.91/2619.37	63
**5**	C_10_H_19_O-O2Oc-[Dap(GO2)]_8_-O2Oc-NH_2_	6.32	2645.96/2647.42	61
**6**	C_12_H_23_O-O2Oc-[Dap(GO2)]_8_-O2Oc-NH_2_	6.92	2674.02/2675.47	52
**7**	C_14_H_27_O-O2Oc-[Dap(GO2)]_8_-O2Oc-NH_2_	7.56	2702.07/2703.27	67
**8**	C_16_H_31_O-O2Oc-[Dap(GO2)]_8_-O2Oc-NH_2_	8.19	2730.10/2731.40	72

*: UPLC analysis (Nexera X2 LC-30AD [Schimadzu, Japan]) equipped with a Phenomenex column (150 mm × 2.1 mm) with a grain size of 1.7 µm (peptide XB-C18), equipped with a UV-Vis detector and a fluorescence detector. A linear gradient from 3% to 90% B within 20 min was applied (A: 0.1% trifluoroacetic acid; B: 80% acetonitrile in A). **: HR MALDI analysis with 2,5-dihydroxybenzoic acid as a matrix. ***: The yield was calculated using the resin substitution (theoretical yield) in relation to the received weight of the final products.

## Data Availability

The original contributions presented in this study are included in the article. Further inquiries can be directed to the corresponding authors.

## Supplementary Materials

The following supporting information can be downloaded at: https://www.mdpi.com/article/10.3390/molecules30071644/s1. Figure S1: UPLC analysis of crude peptides 1–8. The analysis condition: 3-90%B during 20 min, 10 min wash at 90%B, 3 min equilibrated at 3%; Figure S2: Stability assay using NanoDrop assay (Thermo Scientific, Waltham, MA, USA); Table S1: Hydrodynamic diameters and polydispersity index (PDI) of lipidated compounds and DNA plasmid complex.
