# Supplementary material for: Lipidated DAPEG Polymers as a Non-Toxic Transfection Agent—Influence of Fatty Acid Side Chain on Transfection Efficacy
Source: Molecules. 2025 Apr 7;30(7):1644. doi: 10.3390/molecules30071644 (PMC11990301; doi:10.3390/molecules30071644)
Supplement: Supplementary file 1 [file molecules-30-01644-s001.zip › molecules-3485953-supplementary.pdf]

Supplementary Materials

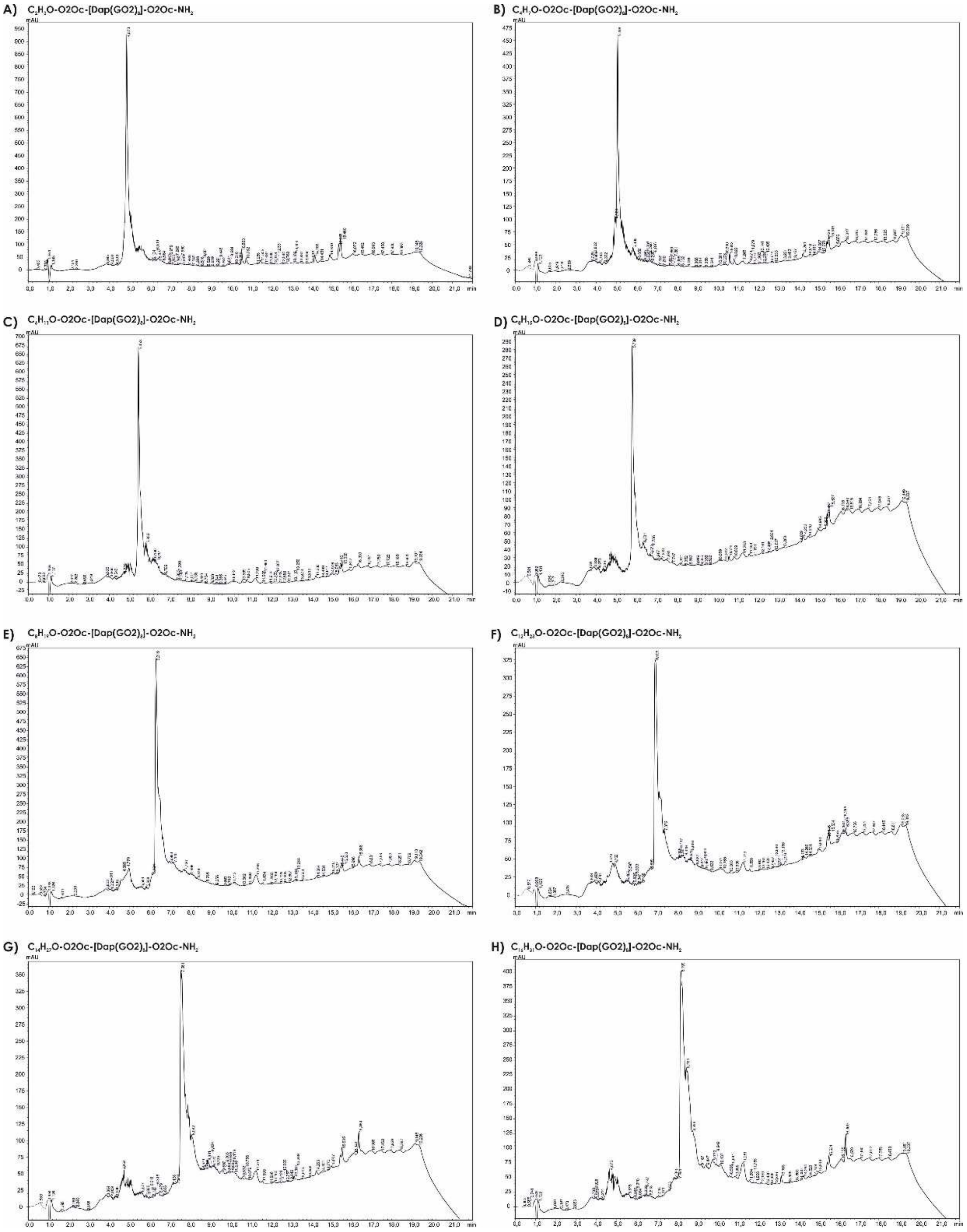

Figure S1. UPLC analysis of crude peptides 1-8. The analysis condition: 3-90%B during 20 minutes, 10 minutes wash at 90%B, 3 minutes equilibrated at 3%.

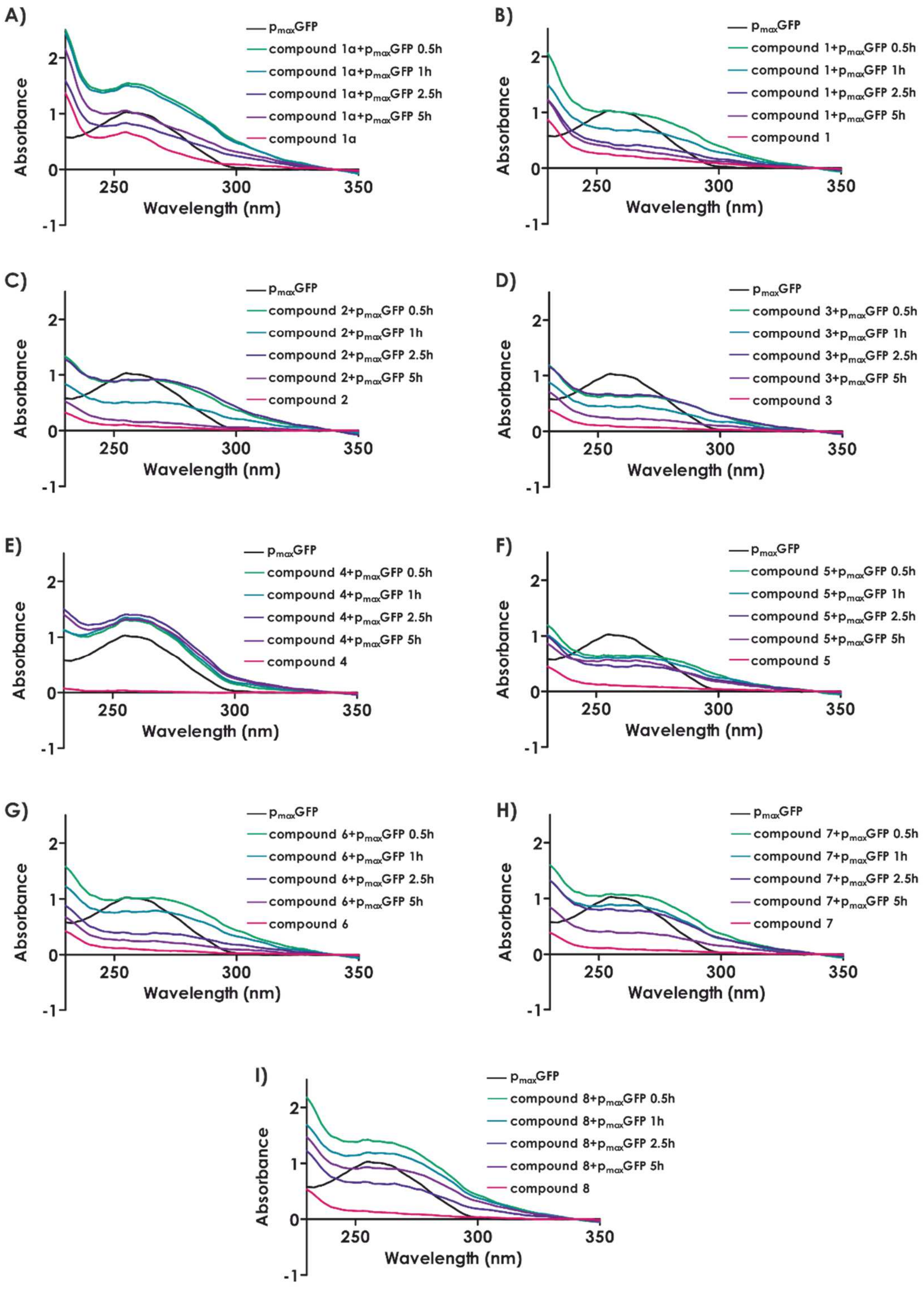

Figure S2. Stability assay using NanoDrop assay (Thermo Scientific, USA).

Table S1. Hydrodynamic diameters and polydispersity index (PDI) of lipidated compounds and DNA plasmid complex.

	Sequence	pMAXGFP (-)		pMAXGFP (+)	
		**hydrodynamic diameters [nm]**	**PDI [%]**	**hydrodynamic diameters [nm]**	**PDI [%]**
**1a.**	H-O2Oc-[Dap(GO2)]$_8$-O2Oc-NH$_2$	329,1 ± 39,3	18,3 ± 1,5	2235,3 ± 48,0	29,9 ± 2,8
**1**	C_2H_3O-O2Oc-[Dap(GO2)]$_8$-O2Oc-NH$_2$	309,0 ± 56,2	25,5 ± 2,9	5580,7 ± 321,2	30,1 ± 1,7
**2**	C_4H_7O-O2Oc-[Dap(GO2)]$_8$-O2Oc-NH$_2$	337,2 ± 28,6	27,8 ± 1,5	4761,0 ± 190,1	30,6 ± 2,5
**3**	$C_6H_{11}O$-O2Oc-[Dap(GO2)]$_8$-O2Oc-NH$_2$	599,5 ± 80,8	23,5 ± 2,4	3006,0 ± 133,9	30,7 ± 2,6
**4**	$C_8H_{15}O$-O2Oc-[Dap(GO2)]$_8$-O2Oc-NH$_2$	508,9 ± 62,2	24,4 ± 2,3	3446,7 ± 208,5	29,4 ± 2,5
**5**	$C_{10}H_{19}O$-O2Oc-[Dap(GO2)]$_8$-O2Oc-NH$_2$	240,6 ± 36,9	24,9 ± 2,2	4605,7 ± 90,9	34,3 ± 4,2
**6**	$C_{12}H_{23}O$-O2Oc-[Dap(GO2)]$_8$-O2Oc-NH$_2$	337,4 ± 17,7	23,2 ± 2,8	283,7 ± 5,9	24,2 ± 1,6
**7**	$C_{14}H_{27}O$-O2Oc-[Dap(GO2)]$_8$-O2Oc-NH$_2$	373,5 ± 59,7	25,6 ± 3,2	135,0 ± 3,2	21,2 ± 2,4
**8**	$C_{16}H_{31}O$-O2Oc-[Dap(GO2)]$_8$-O2Oc-NH$_2$	517,6 ± 63,3	24,8 ± 1,9	308,7 ± 22,7	26,3 ± 2,9
